# A roadmap to implementing outpatient administration of bispecific antibodies in multiple myeloma

**DOI:** 10.3389/fonc.2025.1630146

**Published:** 2025-07-30

**Authors:** Alfred L. Garfall, Rahul Banerjee, Laurent Frenzel, Cyrus Khandanpour, Yi Lin, Erica Ottoni, Robert Rifkin, Sarah Rockwell, Cesar Rodriguez, Humberto Villefort, Elena Zamagni

**Affiliations:** ^1^ University of Pennsylvania, Philadelphia, PA, United States; ^2^ Fred Hutchinson Cancer Center, Seattle, WA, United States; ^3^ Department of Hematology, Hospital Necker, Paris, France; ^4^ University Hospital Schleswig-Holstein, Lübeck, Germany; ^5^ University Cancer Center Schleswig-Holstein, Lübeck, Germany; ^6^ University of Lübeck, Lübeck, Germany; ^7^ Mayo Clinic, Rochester, MN, United States; ^8^ Hospital Moinhos de Vento, Porto Alegre, Brazil; ^9^ US Oncology Research, Rocky Mountain Cancer Centers, Denver, CO, United States; ^10^ Florida Cancer Specialists & Research Institute, Tampa, FL, United States; ^11^ Icahn School of Medicine at Mount Sinai, New York, NY, United States; ^12^ A.C.Camargo Cancer Center, São Paulo, Brazil; ^13^ IRCCS Azienda Ospedaliero-Universitaria di Bologna, Istituto di Ematologia “Seràgnoli”, Bologna, Italy; ^14^ Dipartimento di Scienze Mediche e Chirurgiche, Università di Bologna, Bologna, Italy

**Keywords:** multiple myeloma, bispecific antibodies, bispecifics, outpatient, teclistamab, talquetamab

## Abstract

**Introduction:**

Bispecific antibodies (BsAbs) are novel immunotherapy agents for the treatment of relapsed/refractory multiple myeloma (RRMM). Currently, 3 BsAbs (teclistamab, talquetamab, and elranatamab) are approved for the treatment of RRMM. Administering BsAbs in different practice settings is crucial to improving treatment access and patient outcomes. This report provides actionable guidance to implement safe and effective administration of BsAbs for patients with RRMM in outpatient and community settings.

**Methods:**

Three clinician advisory workshops were held in the United States, Europe, and Latin America to discuss key factors to operationalize BsAb use in outpatient and community settings, focusing on the critical phases of practice setup, treatment initiation, and ongoing management.

**Results:**

BsAb administration in outpatient and community settings requires careful planning, a well-prepared multidisciplinary team (MDT) of healthcare professionals, and clear protocols, including MDT composition, roles/responsibilities, capacity planning, patient selection criteria, step-up dosing procedure, admission processes, patient/caregiver education requirements, and adverse event (AE) monitoring/management. Comprehensive MDT training on protocols and preparedness to manage AEs is essential. Patients initiating outpatient BsAb therapy should have a reliable caregiver, access to a hospital, controlled comorbidities, and no active infections. Ensuring patients and caregivers understand the benefits, risks, and expectations of BsAb therapy is vital for successful treatment and a positive patient experience.

**Conclusion:**

Administering BsAbs in outpatient and community settings can be done safely and effectively with appropriate planning and protocols. Enabling safe and effective BsAb administration in these settings is essential to ensure more patients with RRMM have access to treatment and improved outcomes.

## Introduction

T-cell redirecting bispecific antibodies (BsAbs) are a novel class of immunotherapy agents, with teclistamab, talquetamab, and elranatamab currently approved for the treatment of relapsed/refractory multiple myeloma (RRMM) (1–8). These BsAbs offer single-agent response rates and durations of response comparable with those of chimeric antigen receptor (CAR) T-cell therapies but with the simplicity of an off-the-shelf, subcutaneous injection (4, 5, 9–11). BsAbs are appropriate for many different patients, irrespective of age and frailty (5). As with other immunotherapy agents, adverse events (AEs) associated with BsAbs, including cytokine release syndrome (CRS), immune effector cell–associated neurotoxicity syndrome (ICANS), and risk of infection, must be carefully monitored and managed (4, 5).

Presently, BsAb therapies are primarily administered in hospital and academic settings and US labeling indicates patients should be hospitalized for 24 to 48 hours after step-up dosing (SUD) (6–8). Adoption in other practice settings, such as outpatient clinics and community centers, can be challenging due to a lack of physician experience with BsAbs and necessary infrastructure and resources (4, 12). Developing strategies to implement BsAb use in outpatient and community settings is a critical step toward lowering disparities in RRMM treatment (13) as well as decreasing inpatient hospitalization time, lowering overall healthcare costs, and enhancing the patient experience (12).

This article provides a roadmap for the safe and effective administration of BsAbs in outpatient and community settings, based on the authors’ consensus experience gathered during 3 international workshops held between June 2024 and September 2024 in the United States, Europe, and Latin America.

## Discussion

**Figure 1:**
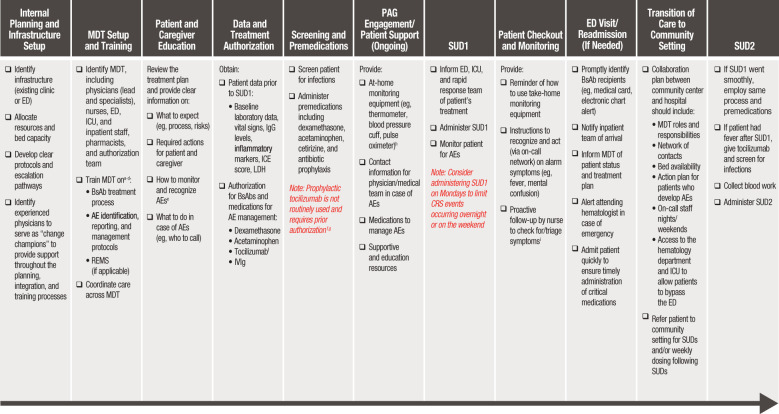
Workflow diagram for BsAb administration in outpatient settings. AE, adverse event; BsAb, bispecific antibody; CRS, cytokine release syndrome; ED, emergency department; ICANS, immune effector cell-associated neurotoxicity syndrome; ICE, immune effector cell encephalopathy; ICU, intensive care unit; IVIg, intravenous immunoglobulin; LDH, lactate dehydrogenase; MDT, multidisciplinary team; MM, multiple myeloma; SUD, step-up dosing. ^a^Physicians should be trained on the mechanism of action of BsAbs and their associated benefits and risks, identification of patients appropriate for BsAb treatment, and prevention/management of AEs. ^b^Implementing a training program with a variety of educational media (eg, live and prerecorded presentations, MDT meetings, continuing medical education, educational posters, cheat sheets) helps provide effective training tailored to various stakeholders. ^c^For MDT members who are not MM specialists, training is an even higher priority. ^d^At minimum, yearly refresher trainings are recommended for all MDT members involved in the BsAb treatment process. ^e^In addition to CRS and ICANS, patients and caregivers should also be informed of the risk of transient neutropenia, infection risk and prevention, and how to recognize infection symptoms. ^f^It may be advisable to include tocilizumab as a prn (as-needed) order within the BsAb treatment plan. ^g^Tocilizumab prophylaxis has been reported to reduce risk of CRS (22–24); however, it does not prevent all CRS events and is not routine practice (25). ^h^If using remote monitoring devices (eg, wearables) to track vitals, provide them to patients ~2 weeks before BsAb administration to allow time for them to become comfortable using the device. ^i^Any fever occurring within the first week should be escalated as it may indicate the onset of CRS; fever occurring later could suggest an infection.

Outpatient administration of BsAbs can be both effective and safe when supported by appropriate infrastructure, a well-prepared multidisciplinary team (MDT) of healthcare professionals, and clear, accessible plans and protocols guiding the entire treatment process—from clinical setup to treatment initiation and ongoing management (14–16). The following practice-based recommendations address the clinical and operational elements necessary to implement BsAb administration safely and effectively in outpatient and community settings. The workflow diagram in **Figure 1** summarizes the key steps and considerations for BsAb administration in outpatient settings.

### Part 1: practice setup for outpatient BsAb administration

#### Infrastructure

Infrastructure-related plans should reflect the type of healthcare center, either an existing outpatient clinic or a new establishment, and if an on-site emergency department (ED) is available for patients in case of serious complications. Outpatient teams should establish a partnership that includes adequate training for their ED team or a hospital to assist with inpatient needs that may arise during treatment.

Upfront bed capacity and resource allocation are important to manage the volume of patients who may be treated at a given time and ensure sufficient beds and availability of on-call care teams for patients requiring admission. Intensive care unit (ICU) capacity should also be secured for care beyond the ED and medical wards.

#### Protocols and logistics

Before introducing BsAbs into a clinical practice, it is essential to develop clear protocols and processes to ensure the effective management of patients, particularly in the early stages of therapy. Protocols related to patient logistics should provide guidance on the requirement of a reliable and informed caregiver—someone who can identify signs and symptoms of AEs, communicate with the medical center/team, and provide/coordinate transportation for the patient. Accommodation plans should be made for patients and caregivers who do not live near a treatment center. Pretreatment blood work, use of prophylactic medications, and admission protocols should be defined. AE management protocols should include detailed guidance on the monitoring, identification, reporting, and management of AEs associated with BsAbs, including CRS, ICANS, and infections, like those published by Mahmoudjafari and colleagues (16).

New protocols may not be required in cases where guidance for BsAb treatment can be integrated into existing processes for other malignancies; for example, CRS management protocols may be embedded within systems already established for CAR T-cell recipients.

#### Pathways for readmission in case of AEs following BsAb administration

An efficient hospital admission pathway should be established to enable patients who experience serious AEs to bypass the ED as appropriate. The pathway should start with a streamlined referral process from the outpatient facility to the hospital, aligned with the hospital’s workflow, to facilitate scheduling and ensure bed availability for patients receiving BsAb treatment. Once the BsAb is administered, the hematologist in charge of treatment should inform the ED, ICU, and rapid response teams to be prepared in case the patient experiences a serious AE. These team members should be well trained in managing BsAb-related AEs. In outpatient clinics that do not operate around the clock, an overnight nursing/physician team should be available for the patient to contact outside of normal business hours. If a patient develops AEs, especially overnight or on a weekend, they may need to visit the ED for care. Essential medications, such as dexamethasone and tocilizumab, must be readily available for CRS management. Proper management of CRS and ICANS is essential to prevent complications.

#### MDT setup and training

A crucial element of successful BsAbs administration is the identification of an MDT including physicians, hematologists, nurses, specialists from the ED, ICU, and infectious disease teams to assist with management of AEs and infection risk, as well as pharmacists and authorization teams who play important roles in treatment access. Protocols should clearly define the roles, responsibilities, and training of MDT members to coordinate care from patient identification through treatment and monitoring.

#### Patient and caregiver education and support during BsAb treatment

Patients and caregivers should have a comprehensive understanding of the benefits, risks, and expectations of BsAb therapy to ensure a positive experience. They must understand AE symptoms to look out for, what to do in case of an AE, and how to use the at-home monitoring equipment. Patient advisory groups can provide additional education and resources, and digital tools may facilitate appointment booking and AE monitoring and reporting.

Patients should receive a take-home package containing direct contact information for the lead physician and medical team, medications for AE management, patient log, AE symptom reminder sheet/card, and monitoring equipment. Medical identification cards can be provided to alert the ED team or other staff that the patient is receiving BsAb treatment.

#### Patient suitability for outpatient BsAb treatment

Most patients with RRMM are eligible to receive BsAbs. Based on the authors’ experience, patients with the following characteristics are likely to be well suited for outpatient treatment: low tumor burden, Eastern Cooperative Oncology Group (ECOG) performance status score of 0 to 2, good mental status, minimal/stable complications of MM disease progression, and well-controlled comorbidities (eg, no history of heart failure or tachyarrhythmia). Close proximity to the hospital and round-the-clock availability of a reliable caregiver is highly recommended. It is important to note that these characteristics represent a near-ideal patient profile and do not preclude patients with higher risk characteristics from being considered for outpatient treatment. The decision to treat a patient in an outpatient setting should be individualized, taking into account potential confounding factors and the overall clinical judgment of the healthcare provider.

#### Administrative and financial considerations

Centers intending to establish outpatient BsAb administration should understand the variability and intricacies of outpatient medication reimbursement. Precertification processes should be implemented to obtain outpatient authorization for BsAb therapy and any additional supportive medications (eg, tocilizumab). Many centers have also implemented “pocket dexamethasone” (also known as “take-home dexamethasone”), whereby patients are prescribed 8 to 10 mg of oral dexamethasone to have on hand (14, 16) for CRS.

### Part 2: initiating BsAb treatment in the outpatient setting

#### Initiating treatment (SUD1) and checkout process

Before treatment, baseline laboratory data (eg, complete blood count with differential, comprehensive metabolic panel, inflammatory markers, lactate dehydrogenase [LDH]) should be obtained along with the patient’s vital signs. Testing for endemic infections, such as cytomegalovirus (CMV), tuberculosis, and hepatitis B, may be recommended where relevant. Treatment is initiated by the infusion staff, administering the BsAb via an SUD schedule, followed by routine patient monitoring until they are cleared for checkout (**Figure 1**).

#### Case study: safe and effective outpatient administration of BsAbs at a university hospital

##### MDT composition and roles

• Hematologists

o Serve as primary physicians for patients
o Select the appropriate treatment
o Conduct patient visits
o Explain treatment to patients and their families
o Write medical reports
o Evaluate toxicities and lab results
o Decide on treatment interruptions or delays
o Respond to calls from patients at home

• Interns/fellows

o Perform the same roles as the hematologists but under the supervision of a senior MD, never independently (except possibly during phone calls)
o Support senior MD

• Data managers

o Order drug to the pharmacy
o Input case report forms (CRFs) if the patient is part of a clinical trial
o Coordinate with the pharmacy regarding the delivery of the drug

• Research nurses

o Administer the drug to patients
o Draw blood samples
o Communicate with patients
o Act as intermediaries between MDs and patients
o Record blood pressure, temperature, and oxygen saturation before drug administration

• Secretaries

o Handle logistical phone calls
o Serve as liaison between MDs and data managers

##### MDT training

- MDT receives training on CRS and ICANS, where these are precisely described, with data from clinical trials and real life shared, and education to recognize each symptom and grading systems.
- All the MDs and nurses are also trained for basic and intermediate life support per hospital rules (training renewed every 2 years via a 2-day dedicated course).

##### Infrastructure

- The outpatient unit has 3 dedicated rooms from 8:00 AM to 2:00 PM daily, where patient visits occur, and MDs receive phone calls. Each room has a bed, a table, 2 chairs for patient and relatives, and 2 chairs for the senior and junior MDs. Patients receiving BsAbs are scheduled for visits during this timeframe, integrated with the visits of other patients being managed by the same medical team.
- There are also dedicated rooms for drug injections and blood samples, where the secretary and the nurse are present. These rooms have emergency equipment (eg, defibrillator and drugs) as well as blood pressure and oxygen saturation measurement devices.

##### Patients treated

- Among 10 patients receiving BsAb treatment, 7 were eligible for outpatient administration based on caregiver availability, comorbidity burden, disease stage, and distance from hospital.

##### Prophylaxis in preparation for SUD1

- Dexamethasone (20 mg) administered 1 to 2 hours before BsAb on Days 1 and 2 to 4.
- Acetaminophen and cetirizine given on Days 1 and 3.
- One patient received tocilizumab 2 hours before BsAb treatment.

##### SUD1

- Patients stayed in a dedicated waiting area for 4 hours after SUD1 only.
- Patients were sent home with instructions to contact the MDT or the hematologist on call if they experienced fever >100.4°F (38°C), breathing issues, or any clinical issue (eg, chills, cough, chest or abdominal pain) and to go to the ED in case of serious complications.
- Take-home medications included dexamethasone, acetaminophen, and cetirizine.
- Patients were instructed to contact the MDT if fever occurred after Day 4.
- IVIg was administered if IgG level was <400 mg/dL.

##### Follow-up

- Patients were scheduled to visit the hospital on Days 1, 3, and 8.
- Subsequent visits were based on the BsAb administration schedule and the occurrence of complications.

##### Outcomes of 7 patients with MM treated with BsAbs in the outpatient setting

- Two patients experienced grade 1 CRS on Day 8, which was managed with an additional dose of dexamethasone.
- Three patients had persistent mild fever (99.5°F [37.5°C]) during the first 5 to 8 injections, which was treated with an additional 20 mg dose of dexamethasone after each BsAb dose.
- Three patients developed upper respiratory tract infections at 2, 4, and 7 weeks after SUD1, which were managed according to standard of care with antibiotics, when necessary, and mucolytic drugs.

##### Learnings

- Physicians should be well prepared and confident in their procedures for administering BsAbs.
- The MDT should collaborate effectively.
- Patients should have a robust support network at home.
- Patients need easy and reliable ways to contact their physicians.
- Each center should have a structured learning curve; as experience is gained, the treatment process improves, leading to reduced anxiety and better coordination among different roles.

#### Note on generalizability

While this case study provides a detailed example from a university hospital setting, the principles and strategies outlined can be adapted to various healthcare environments. Each center should consider its unique resources, infrastructure, and patient population when developing its protocols for outpatient BsAb administration. The goal is to ensure safety, efficacy, and patient support regardless of the specific setting.

### Part 3: ongoing patient management after BsAb SUD

Ongoing management focuses mainly on preventing and monitoring for typical/opportunistic infections. Risk factors for infection in patients with RRMM include multiple lines of treatment for the disease, immunosuppressive therapies to manage AEs, neutropenia, and hypogammaglobulinemia (17). Opportunistic infections (eg, CMV, pneumocystis jiroveci pneumonia, and aspergillosis) can be severe, and BsAb therapy should not be initiated if the patient has an active infection (18). Infection prophylaxis should consider patient and disease-related factors, including comorbidities, cytopenias, prior therapy, and prior infections (16, 19), and consultation with infectious disease specialists. Antibiotics, antifungals, or antivirals should be considered at the onset of BsAb treatment if neutropenia exists or, at minimum, in the first 2 to 3 months of treatment. IgG levels should be monitored routinely and IVIg therapy should be initiated if levels drop below 400 mg/dL or if patients have severe/recurrent infections (19–21).

Talquetamab is associated with dysgeusia, dry mouth, dysphagia, and AEs that affect the skin and nails (5, 10). Oral-related AEs can be managed with supportive care and dose modifications. Skin-related AEs of exfoliation, pruritus, and dry skin can be managed with topical and oral glucocorticoids.

**Figure 2:**
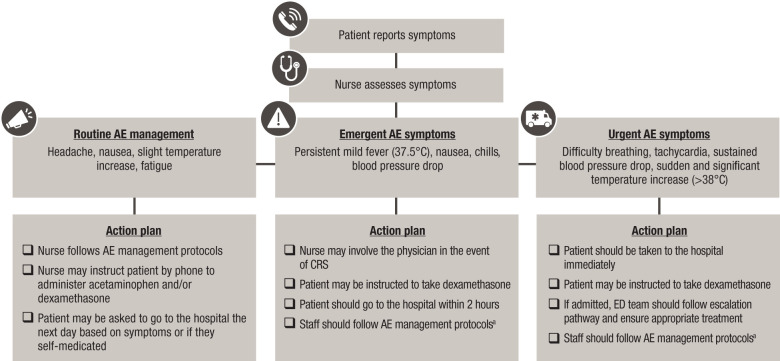
Management of AE symptoms following BsAb administration in the outpatient setting. AE, adverse event; BsAb, bispecific antibody; CRS, cytokine release syndrome; ED, emergency department. Note: Action plans are center specific and informed by infrastructure, resources, and familiarity with BsAb therapies. ^a^In line with current International Myeloma Working Group consensus guidelines, tocilizumab is recommended for the treatment of grade 1 CRS, and early intervention (at first sign of fever) is encouraged (25).

Nurses can facilitate triage of AE symptoms by severity and follow protocols to manage AEs or escalate AE management to the physician while providing clear instructions to the patient on when to go to the hospital (**Figure 2**).

### Transition of care to community settings

Referrals from community practices for outpatient BsAb administration should be done in partnership with academic centers with established experience with BsAb therapy. After SUD, patients may transition back to a community center for treatment maintenance (**Figure 1**).

## Conclusions

Safe and effective administration of BsAbs in outpatient and community settings is feasible with appropriate planning and process development. Centers can start slowly, gaining experience with patients in the hospital setting and then moving patients to an established outpatient setting.

Critical steps for transitioning to outpatient administration of BsAbs include:

- Establishing capacity rules.
- MDT setup and training.
- Developing protocols and escalation pathways.
- Building relationships between academic and community centers.
- Coordinating care between these centers.

While these recommendations provide a practical roadmap for implementing BsAb administration in outpatient and community settings, some limitations persist, as not all centers will have adequate staffing, equipment, or infrastructure. However, enabling more centers to adopt these practices is critical to increase the accessibility of care and improve outcomes for patients with RRMM.

## Data Availability

The raw data supporting the conclusions of this article will be made available by the authors, without undue reservation.
